# Mapping QTLs for Fertility Restoration of Different Cytoplasmic Male Sterility Types in Rice Using Two* Oryza sativa ×O. rufipogon* Backcross Inbred Line Populations

**DOI:** 10.1155/2016/9236573

**Published:** 2016-10-31

**Authors:** Biao-lin Hu, Jian-kun Xie, Yong Wan, Jin-wei Zhang, Fan-tao Zhang, Xia Li

**Affiliations:** ^1^College of Life Science, Jiangxi Normal University, Nanchang, Jiangxi 330022, China; ^2^Rice Research Institute, Jiangxi Academy of Agricultural Sciences and Nanchang National Sub-Center for Rice Improvement, Nanchang, Jiangxi 330200, China

## Abstract

Hybrid rice breeding using cytoplasmic male sterility/fertility restoration (CMS/*Rf*) systems plays an important role in ensuring global food security. Two backcross inbred line (BIL) populations derived from either Xieqingzao B (XB)//XB/Dongxiang wild rice (DWR) (XXD) or XB//DWR/XB (XDX) were used to detect quantitative trait loci (QTLs) for fertility restoration of Dwarf wild abortive- (DA-), Indonesia Paddy- (ID-), and DWR-type CMS in rice. Lines with ID- and DA-type CMS were testcrossed with both the XXD- and XDX-BILs, while the line with DWR-type CMS was testcrossed with the XDX-BILs only. A total of 16 QTLs for fertility restoration of CMS systems were identified, including three for DWR-type CMS, six for DA-type CMS, and seven for ID-type CMS. All of the additive alleles in the QTLs were derived from* Oryza rufipogon*. Eleven QTLs were clustered in five chromosomal regions, indicating that common* Rf* loci restored different CMS systems, and the favorable* O*.* rufipogon* alleles could be used to develop restorer lines for various CMS types by marker-assisted selection.

## 1. Introduction

Rice (*Oryza sativa *L.) is one of the most important staple crops worldwide, providing 21% of the food calorie supply for the world's population and up to 76% of that for Southeast Asia [1]. Thus, the improvement of global food security relies heavily on the ability to sustainably increase rice yields [2, 3]. Such increases are possible through the use of novel high-yielding varieties of rice, which will depend in turn on the development of heterotic rice hybrids [4].

Hybrids often exhibit superior growth traits than either parental line, due to what is referred to as heterosis or hybrid vigor [5]. Cytoplasmic male sterility/fertility restoration (CMS/*Rf*) systems provide a powerful approach in the production of hybrid seed [6]. CMS/*Rf* systems are widespread in flowering plant species. CMS caused by an incompatibility between mitochondrial and nuclear genomes results in unviable pollen, preventing seed development in a self-pollinating plant like rice. CMS can however be rescued by nuclear* Rf* gene(s) [7–9]. Therefore, in rice breeding programs, the combination of CMS in the female parent and* Rf *gene(s) in the male parent is indispensable for the commercial development of three-line hybrid rice.

Since the initial discovery of CMS in a rice line that possessed the Chinsurah Boro II cytoplasm, over 60 types of CMS associated with different cytoplasmic resources have been reported [10]. In rice, thirteen types of CMS have been commercially used for hybrid rice production, namely, those from Boro II (BT), Dwarf wild abortive (DA), Dissi (D), Dian1 (Dian1), Gambiaka (G), Honglian (HL), Indonesia Paddy (ID), K52 (K), Luihui (LX), Maxie (Maxie), NX (NX), wild abortive (WA), and Yegong (Y) [11]. Although the use of WA-type CMS is still dominant, there has been a gradual increase in the proportion of ID- and DA-type CMS used for hybrid rice production [11, 12]. However, relatively little is known regarding the origins, evolutionary relationships, and distribution of* Rf* genes for ID- and DA-type CMS.

In general, CMS can be restored by one or two nuclear* Rf* genes. For example, in WA-type CMS, pollen abortion caused by the* WA352 *gene is rescued by nuclear genes* Rf3* and* Rf4*, which are located on chromosomes 1 and 10, respectively [8]. A large number of reports have focused on the mapping of* Rf* genes or loci that rescue various CMS types, such as those from BT, Chinese wild rice (CW), D, DA, Dian1, G, HL, Lead Rice (LD), ID, and WA [13–19]. In these studies,* Rf* genes were detected by bulk segregant analysis of an F_2_ or backcross population. To date, more than 17* Rf *genes or loci that rescue different CMS types have been identified, distributed across all rice chromosomes apart from chromosome 9 (Gramene, http://www.gramene.org). Of these, seven* Rf *genes have been identified and functionally characterized:* Rf1 *(*Rf1a* and* Rf1b*) for BT-type CMS [20, 21],* Rf2 *for LD-type CMS [22],* Rf4 *for WA-type CMS [23, 24],* Rf5 *and* Rf6 *for HL-CMS [25, 26], and* Rf17 *for CW-CMS [27]. Molecular cloning and characterization of these* Rf *genes not only further described the cytoplasmic-nuclear interactions, but also provided powerful molecular tools to assist in hybrid breeding by accelerating the development of novel restorer lines. However,* Rf7* to* Rf16 *remain to be characterized, suggesting that further study is needed on the genetic effect of* Rf* genes [28]. Furthermore, although five of the seven characterized* Rf* genes (all except* Rf2 *and* Rf17*) are used in commercial hybrid rice production in China, they all originate from* indica* rice. The current limit in yield from three-line hybrid rice in China might be explained by the small number of* Rf* genes used to develop restorer lines in hybrid rice breeding [12]. Thus, exploration of new* Rf* genes will greatly enrich the resources available to improve rice yields using heterosis. Furthermore, the exact number of different* Rf* genes that exist for a particular CMS type is still unclear. Therefore, the discovery of new* Rf* loci or alleles in wild rice not only will add to the current genetic resources used in CMS/*Rf* systems for hybrid rice, but also will lead to a better understanding of the origin and evolution of* Rf* genes in rice.

Dongxiang wild rice (*Oryza rufipogon *Griff., hereafter referred to as DWR) is a common wild rice variety distributed in northern regions worldwide. In addition to its remarkable traits for cold and drought tolerance [29, 30], DWR carries* Rf* genes for CMS [31]. Therefore, there is potential to identify these valuable* Rf* genes and develop them for use in hybrid rice breeding. In the present study, an interspecific cross between a rice cultivar and an accession of DWR was conducted to detect QTLs for fertility restoration of various CMS types. The objective of this work was to identify potential alleles from* O*.* rufipogon *to add to the* Rf *genes currently used in CMS/*Rf* systems for hybrid rice.

## 2. Materials and Methods

### 2.1. Plant Materials

Two mapping populations consisting of either 202 or 237 backcross inbred lines (BILs) were derived from a backcross using rice cultivar Xieqingzao B (*Oryza sativa* L., hereafter referred to as XB) as the recurrent parent and an accession of DWR as the donor parent. The development process of 202 BC_1_F_5_ BILs derived from cross XB//XB/DWR (hereafter referred to as XXD) was described by Chen et al. [32]. The 237 BC_1_F_10_ BILs were established by first crossing an accession of DWR as the female parent with cultivated rice XB to generate an F_1_ hybrid in 1998 by Rice Research Institute, Jiangxi Academy of Agricultural Sciences (RRI, JAAS), Nanchang, China. Following this, the F_1_ plants were backcrossed to XB to generate a BC_1_F_1_ cross of XB//DWR/XB (hereafter referred to as XDX), from which a BIL population of 237 BC_1_F_10_ lines was obtained by single seed descent in 2008.

A controlled cross between three alloplasmic CMS lines, including Xieqingzao A (XA), Zhong 9A (ZA), and DongB11 A (DbA), and each line of the BC_1_F_5_ XXD-BIL and BC_1_F_10_ XDX-BIL population was made, forming five sets of the testcross populations. As a result, two and three sets of the F_1_ testcross populations (XA/XXD-BIL and ZA/XXD-BIL, and DbA/XDX-BIL, XA/XDX-BIL, and ZA/XDX-BIL) were obtained in 2010 and 2014, respectively. Xieqingzao A is a typical DA-type CMS line of superhybrid rice Xieyou9308. Zhong 9A is a typical ID-type CMS line of superhybrid rice Zhongyou228. DongB11 A is a CMS line with DWR cytoplasm of superhybrid rice Dongyou 962, which was bred by Xiao et al. [33].

### 2.2. Field Experiments and Phenotyping

In the rice-growing seasons from May to October in 2010 and 2014, the phenotyping experiments were conducted in experimental fields at the Rice Research Institute, Jiangxi Academy of Agricultural Sciences in Nanchang, Jiangxi (latitude: 28°33′N, longitude: 115°56′E). In 2010 experiment, two sets of testcross populations consisting of 202 XA/XXD-BIL and ZA/XXD-BIL lines and the F_1_ progeny from crosses between XB and two CMS lines were grown. In 2014 experiment, three sets of testcross populations consisting of 237 DbA/XDX-BIL, XA/XDX-BIL, and ZA/XDX-BIL lines and the F_1_ progeny from crosses between parent XB and three CMS lines were grown. In field trials, each line was transplanted to plots with a spacing of 16.7 cm between plants within a row and 26.7 cm between rows. Each plot consisted of three rows with 12 plants per row. The field trial was managed with the normal agricultural practice.

At maturity, five plants from the middle of each line plot were harvested, unless high sterility was observed, in which case only one plant was harvested. For phenotyping of the fertility restoration, spikelet fertility (SF) was estimated as the ratio (percentage) of filled grains to the total number of grains per panicle. The average SF value of the randomly sampled plants from each line was used as a measure of fertility restoration for data analysis.

### 2.3. DNA Marker Analysis and Genetic Map Construction

The 237 BC_1_F_10_ BILs and the recurrent parent XB were grown in 2009 in Nanchang. The fresh leaves from a single plant per line were collected for total genomic DNA extraction according to Zheng et al. [36]. A total of 300 SSR and 50 InDel markers were used to survey parental polymorphism. All of SSR markers were obtained from GRAMENE (http://www.gramene.org). The InDel markers were designed according to the DNA length polymorphism of Nipponbare and 93-11 (https://www.ncbi.nlm.nih.gov/) using Premier 5.0 software. As the original DWR accession is no longer available, three DNA bulks, each consisting of 10 randomly selected BILs, and the DNA from the parent XB were used.

Polymerase chain reaction (PCR) was performed in a 10 *μ*L reaction mixture containing 5.0 *μ*L of 2 Taq Master Mix (CWBIO, Beijing, China), 3 MlRnase-free H_2_O (CWBIO, Beijing, China), 1 *μ*L template DNA, and 1 *μ*L of 3.3 ng *μ*L^−1^ SSR primers. The cycling profile for the PCR consisted of initial denaturation at 94°C for 2 min, 30 cycles at 94°C for 30 s, 55°C for 30 s, and 72°C for 30 s, followed by a final extension at 72°C for 2 min. The PCR products were separated on 2.5% agarose gel stained with Gelred (Biotium, Hayward, CA, USA) and recorded using a MiniBIS Pro (DNR Bio-Imaging Systems Ltd., Jerusalem, Israel) or 6% nondenaturing polyacrylamide gel and silver staining, depending on the sizes of the DNA fragments and clarity of the bands [36].

### 2.4. Linkage Map Construction and Data Analysis

Linkage map for the XXD population was firstly constructed by Chen et al. [32] and updated by Huang et al. [37]. For the XDX population, each BIL was genotyped with 145 polymorphic markers, including 12 InDels and 133 SSRs. The linkage map was constructed using Mapmaker/Exp 3.0 software [38]. Distances between markers were estimated using the Kosambi function and presented in centiMorgan (cM).

Descriptive statistics of phenotypic data, including minimum and maximum trait values, mean trait value, standard deviation, coefficient of variation, skewness, and kurtosis, were calculated using the command DSum of the software Windows QTL Cartographer 2.5 [39].

QTLs for fertility restoration were detected using Windows QTL Cartographer 2.5 [39]. Composite interval mapping was performed using a walking speed of 1 cM and a window size of 10 cM with backward and forward regression. A logarithm of the odds (LOD) threshold > 2.0 was used to claim a putative QTL. The QTL was designated following the nomenclature commended by McCouch and CGSNL [40].

## 3. Results

### 3.1. Phenotypic Performance

Descriptive statistics of spikelet fertility are summarized in Table 1. Wide variations of spikelet fertility with continuous distribution were observed in each of the five F_1_ testcross populations.

For the F_1_ testcross population sets grown in 2010, average spikelet fertility of the testcross population ZA/XXD was higher than that of the XA/XXD population. Positive correlation with moderate coefficients of 0.606 (*P* < 0.01) was observed between the test populations. For the F_1_ testcross population sets grown in 2014, average spikelet fertility in the testcross population XA/XDX was highest, followed by lower spikelet fertility in the ZA/XDX and lower still in the DbA/XDX population. Highly significant (*P* < 0.01) and positive correlations were shown between DbA/XDX-BIL and ZA/XDX-BIL populations, DbA/XDX-BIL and XA/XDX-BIL populations, and ZA/XDX-BIL and XA/XDX-BIL populations, with coefficients of 0.611, 0.596, and 0.554, respectively.

### 3.2. Construction of Linkage Map

Of the 300 SSR and 50 InDel markers examined, 135 SSR and 18 InDel markers were shown to be polymorphic, for a ratio of 43.71%, and used for genotyping of the 237 XDX-BILs. A linkage map of the XDX population was constructed using 133 SSRs and 12 InDels and spanned 1620.9 Cm of the 12 rice chromosomes with an average distance of 11.2 cM between adjacent markers.

### 3.3. QTLs Determination

**Figure 1:**
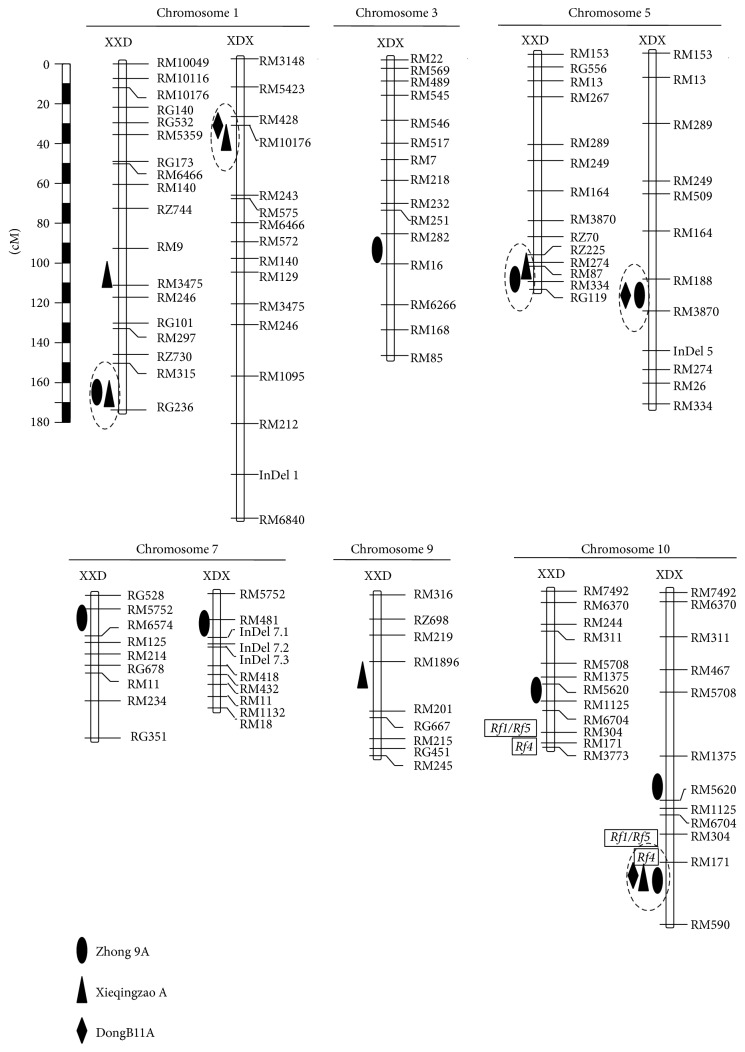
Chromosomal positions of the QTLs conferring fertility restoration for DA-, DWR-, and ID-type CMS. XXD and XDX represent the backcross inbred lines of Xieqingzao (XB)//XB/Dongxiang wild rice (DWR) and XB//DWR/XB, respectively.

A total of 16 QTLs for fertility restoration of three types of CMS were detected, including three for DWR-type CMS, six for the DA-type CMS, and seven for the ID-type CMS (Table 2 and Figure 1). They were distributed on rice chromosomes 1, 3, 5, 7, 9, and 10.* qRf7* and* qRf10.1 *for ID-type CMS were found in the interval RM5752-RM6574 on chromosome 7 and in the vicinity of RM5620 on chromosome 10 in the XDX- and XXD-BIL, respectively. The proportion of phenotypic variance explained (*R* ^2^) by a single QTL ranged as 11.7%–54.9% for DWR-type CMS, 8.2%–49.3% for DA-type CMS, and 4.9%–44.3% for ID-type CMS, respectively.

In the F_1_ testcross population of DbA/XDX-BIL, three QTLs for fertility restoration were detected. Individually, these QTLs explained 11.7%–54.9% of the phenotypic variance and had additive effects ranging from 8.8% to 17.2%. The trait-enhancing alleles were all from DWR. Of these, the QTL* qRf10.2 *had the largest LOD score of 22.78 and contributed 54.9% to the phenotypic variance, with the DWR allele increasing spikelet fertility by 17.2%.

For DA-type CMS, six QTLs were detected for fertility restoration, including two detected in the XA/XDX-BILs population and four in the XA/XXD-BIL population. For* qRf5.1*, which had the lowest *R* ^2^ of 8.2%, the DWR alleles increased spikelet fertility by 5.3%. For the remaining five QTLs, which had *R* ^2^ ranging from 15.1% to 49.3%, the DWR alleles improved spikelet fertility by 6.1%–17.8%.

For ID-type CMS, seven QTLs were detected for fertility restoration, including two detected in both the ZA/XDX-BIL and ZA/XXD-BIL, three detected in the ZA/XDX-BIL only, and two detected in the ZA/XXD-BIL only. The common QTL* qRf7 *had LOD scores of 3.28 and 3.13 in the ZA/XDX-BIL and ZA/XXD-BIL, with the DWR alleles increasing spikelet fertility by 11.3% and 6.9%, respectively. The other common QTL* qRf10.1 *had LOD scores of 3.70 and 6.30 in the ZA/XDX-BIL and ZA/XXD-BIL, with the* O*.* rufipogon* alleles enhancing spikelet fertility by 9.1% and 6.2%, respectively. The remaining QTLs were* qRf3*,* qRf5.1*, and* qRf10.2*, detected in the ZA/XDX-BIL, and* qRf1.4 *and* qRf5.2* detected in the ZA/XXD-BIL. Individually, these QTLs contributed 4.9%–44.3% of the phenotypic variance and had additive effects ranging from 4.0% to 17.2%. The trait-enhancing alleles were again all derived from DWR.

## 4. Discussion

The exploitation of heterosis, such as by using F_1_ hybrids, is one of the most significant genetic achievements in agriculture. Nuclear* Rf* genes complement the male-sterile phenotype that arises with CMS, allowing the use of the CMS system for hybrid seed production. Molecular mapping of the* Rf* genes provides a powerful tool to develop restorer lines avoiding extensive testcrossing with CMS lines [41]. In this study, QTL analysis for fertility restoration of the DA-, DWR-, and ID-type CMS systems was conducted using two* O*.* sativa* ×*O*.* rufipogon* BIL populations. In total, 16 QTLs were detected, three for DWR-type CMS, six for DA-type CMS, and seven for ID-type CMS. It is notable that all 16 QTLs detected for fertility restoration of DA-, DWR-, and ID-type CMS had the enhancing alleles derived from DWR. The use of the favorable* O*.* rufipogon *QTL alleles would facilitate breeding of restorer lines in hybrid rice breeding.

Six of the QTLs detected in this study were located in genomic regions where QTLs for fertility restoration of the same type of CMS have been reported, including three of the six QTLs for fertility restoration of DA-type CMS and three of the seven QTLs for fertility restoration of ID-type CMS (Table 2). For DA-type CMS,* qRf1.2 *colocated with a QTL for fertility restoration reported by Xie et al. [34];* qRf10.2* colocated with a QTL for spikelet fertility reported by Xie et al. [19, 34], which also corresponded to* Rf3* and* Rf4 *for WA-type CMS [42–46], and* qRf5.2* overlapped with* qRf-5* identified by Xie et al. [19]. For ID-type CMS,* qRf1.4* colocated with* qRf5 *reported by Shen et al. [35];* qRf10.1 *and* qRf10.2 *located to both sides of* Rf-4* detected by Li et al. [14], which is also involved in restoring fertility of WA-type CMS. Recently, the gene* Rf4*, which restores WA-type CMS near the QTL* qRf10.2* (Figure 1) and encodes a pentatricopeptide repeat-containing protein, has been cloned [23, 24]. Thus, it may be considered that these QTLs are consistently detected across different environments and genetic backgrounds. In addition,* qRf1.3*,* qRf3*,* qRf7*,* qRf9*, and* qRf5.1 *did not colocate with QTLs previously reported for fertility restoration of other CMS types and could be considered as new fertility restoration loci. These QTLs provide not only good candidates for QTL fine-mapping and cloning, but also novel genetic source for the breeding of restorer line in rice.

Similar to the phenomena reported in many other studies, clustering of QTLs for fertility restoration was evident in the present study. Eleven of the QTLs detected in present study were located in five clusters distributed on three chromosomes (Figure 1). QTLs conferring fertility restoration for DA-, DWR-, and ID-type CMS were located in interval RM171-RM590 on chromosome 10. QTLs conferring fertility restoration for two CMS types were located in each of the other clusters, including two clusters each on chromosomes 1 and 5. These* Rf* loci clusters are of great importance for developing sustainable hybrid rice because they allow for the use of diverse CMS cytoplasmic sources and thus avoid the genetic vulnerability of monoculture in rice agriculture. QTL clusters* qRf1.1* for DA- and DWR-type CMS;* qRf1.4 *and* qRf5.2 *for DA- and ID-type CMS; and* qRf10.2 *for three CMS types were clustered in four regions that have been previously reported (Table 2). Therefore, particular attention should be given to the QTL cluster* qRf5.1* in future studies of QTL fine-mapping and cloning.

The XXD BILs were previously conducted for mapping of QTLs for grain yield traits [37]. Of the 23 QTLs for yield components detected by Huang et al. [37],* qTNSP9* was located in the region corresponding to* qRf9*, and the allelic direction for fertility restoration was identical to the total number of spikelets per panicle. It was suggested that the* Rf *locus* qRf9 *might contribute to grain yield in rice.

Interestingly, major* Rf* genes for other CMS systems in rice, such as* Rf1 *for BT-type CMS,* Rf4 *for WA-type CMS,* Rf-D1(t) *for Dian1-type CMS,* Rf5 *for HL-type CMS, and* qRf-10-2 *for DA-type CMS, were detected on the same region of chromosome 10 [17, 19, 25, 45–47] and were closely linked to form a gene cluster. Cloning of the* Rf* genes and sequence analysis reported by Tang et al. [24] and Hu et al. [25] revealed that the fertility restorer locus* Rf1a *was identical to the locus* Rf5 *but different from the locus* Rf4*. In the present study, the major QTL* qRf10.2 *for three CMS types was located in the same chromosomal region as* Rf4*. Further studies are required to define the allelic relationship between these genes.

For both DWR- and DA-type CMS, the genetic effect of* qRf10.2 *was more pronounced than that of* qRf1.1*. Similar results were reported for WA-type CMS in that the effect of* Rf4* appeared to be larger than that of* Rf3* and inheritance of both* Rf* genes resulted in compounded effects [18, 46, 48, 49]. The mitochondrial gene* WA352 *was recently described in wild rice and is associated with WA-, ID-, and DA-type CMS, suggesting that* Rf3* (*qRf1.1*) and* Rf4* (*qRf10.2*) are effective for restoring the fertility of DA-, ID-, and WA-type CMS [8]. This indicates that the two major* Rf* loci are associated with the different CMS/*Rf* systems in DA-, DWR-, and WA-type CMS. These results should lead to positive outcomes from alloplasmic line breeding and the introduction of DWR* Rf *genes into cultivated rice using advanced backcross QTL analysis in hybrid rice breeding programs.

When QTL results from the same mapping population were compared, the number and genetic effects of the identified QTLs varied amongst three CMS types, suggesting that different* Rf* alleles interact with each form of CMS in an independent manner. This study has provided novel information on the effects of* O*.* rufipogon* QTL alleles (*qRf1.1*,* qRf5.1, *and* qRf10.2*) in DWR-type CMS. The results are in agreement with those reported by Li et al. [31], who claimed that* Rf* alleles coevolve with CMS. That is, wild rice lines that exhibit CMS may also carry the most useful genetic resources for associated fertility restoration. The* O*.* rufipogon* QTL alleles identified here provide good candidate* Rf *genes that can be used to develop new restorer lines for hybrid rice breeding and also further our understanding of the origin and evolution of* Rf *genes in rice.

## Figures and Tables

**Table 1:** Performance of spikelet fertility in the testcross populations and F_1_ plants from Xieqingzao B (XB) crossed with CMS lines.

Year	Combination	F_1_ fertility^a^ (%)	Testcross population					
			Mean (%)	SD	CV	Range (%)	Skewness	Kurtosis
2010	ZA/XXD-BIL	5.1	17.5	13.7	0.78	1.1–90.4	2.0	6.0
	XA/XXD-BIL	0.6	15.0	12.9	0.86	1.4–82.6	2.6	9.2
2014	ZA/XDX-BIL	7.5	35.7	22.26	0.62	1.8–88.9	0.55	−0.81
	XA/XDX-BIL	1.1	38.8	23.46	0.60	3.0–93.2	0.46	−0.87
	DbA/XDX-BIL	20.0	34.9	22.09	0.63	2.6–89.2	0.44	−0.92

^a^F_1_ fertility means the average F_1 _fertility derived from parent Xieqingzao B and CMS lines (ZA, XA, and DbA).

**Table 2:** QTLs conferring fertility restoration for DA-, DWR-, and ID-type CMS detected in the two sets of BIL populations.

Population	QTL	Interval	LOD	*A*	*R* ^2^	Previous reports
DbA/XDX-BIL	*qRf1.1*	RM428-RM10176	7.63	11.6	13.2	
	*qRf5.1*	RM188-RM3870	4.84	8.8	11.7	
	*qRf10.2*	RM171-RM590	22.78	17.2	54.9	
XA/XDX-BIL	*qRf1.2*	RM10176-RM243	6.65	17.6	38.9	[19]
	*qRf10.2*	RM171-RM590	11.59	17.8	49.3	[19, 34]
XA/XXD-BIL	*qRf1.3*	RM9-RM3475	4.86	6.1	15.1	
	*qRf1.4*	RM315-RG236	9.00	19.7	30.2	
	*qRf5.2*	RM87-RM334	4.28	5.3	8.2	[19]
	*qRf9*	RM1896-RM201	2.28	6.8	16.0	
ZA/XDX-BIL	*qRf3*	RM282-RM16	4.17	10.9	14.9	
	*qRf5.1*	RM188-RM3870	5.59	11.1	17.4	
	*qRf7*	RM481-Indel 7.1	3.28	11.3	9.3	
	*qRf10.1*	RM1375-RM5620	3.70	9.1	13.2	
	*qRf10.2*	RM171-RM590	13.33	17.2	44.3	[14]
ZA/XXD-BIL	*qRf1.4*	RM315-RG236	6.58	14.9	27.5	[35]
	*qRf5.2*	RM334-RG119	2.34	4.0	4.9	
	*qRf7*	RM5752-RM6574	3.13	6.9	10.0	
	*qRf10.1*	RM5620-RM1125	6.30	6.2	14.0	[14]

*A* indicates an additive effect of replacing a Xieqingzao B allele by a Dongxiang wild rice allele; *R* ^2^ indicates the proportion of phenotypic variance explained by the QTL effect.
